# Impact of the Genetic Background on the Composition of the Chicken Plasma MiRNome in Response to a Stress

**DOI:** 10.1371/journal.pone.0114598

**Published:** 2014-12-04

**Authors:** Marie-Laure Endale Ahanda, Tatiana Zerjal, Sophie Dhorne-Pollet, Andrea Rau, Amanda Cooksey, Elisabetta Giuffra

**Affiliations:** 1 INRA, UMR 1313 Génétique Animale et Biologie Intégrative, Jouy-en-Josas, France; 2 CEA, DSV, IRCM, SREIT, Laboratoire de Radiobiologie et Etude du Génome, Jouy-en-Josas, France; 3 AgroParisTech, UMR 1313 Génétique Animale et Biologie Intégrative, Paris, France; 4 School of Animal and Comparative Biomedical Sciences, University of Arizona, Tucson, Arizona, United States of America; 5 BIO5 Institute, University of Arizona, Tucson, Arizona, United States of America; University of California, Davis, United States of America

## Abstract

Circulating extra-cellular microRNAs (miRNAs) have emerged as promising minimally invasive markers in human medicine. We evaluated miRNAs isolated from total plasma as biomarker candidates of a response to an abiotic stress (feed deprivation) in a livestock species. Two chicken lines selected for high (R+) and low (R−) residual feed intake were chosen as an experimental model because of their extreme divergence in feed intake and energy metabolism. Adult R+ and R− cocks were sampled after 16 hours of feed deprivation and again four hours after re-feeding. More than 292 million sequence reads were generated by small RNA-seq of total plasma RNA. A total of 649 mature miRNAs were identified; after quality filtering, 148 miRNAs were retained for further analyses. We identified 23 and 19 differentially abundant miRNAs between feeding conditions and between lines respectively, with only two miRNAs identified in both comparisons. We validated a panel of six differentially abundant miRNAs by RT-qPCR on a larger number of plasma samples and checked their response to feed deprivation in liver. Finally, we evaluated the conservation and tissue distribution of differentially abundant miRNAs in plasma across a variety of red jungle fowl tissues. We show that the chicken plasma miRNome reacts promptly to the alteration of the animal physiological condition driven by a feed deprivation stress. The plasma content of stress-responsive miRNAs is strongly influenced by the genetic background, with differences reflecting the phenotypic divergence acquired through long-term selection, as evidenced by the profiles of conserved miRNAs with a regulatory role in energy metabolism (gga-miR-204, gga-miR-let-7f-5p and gga-miR-122-5p). These results reinforce the emerging view in human medicine that even small genetic differences can have a considerable impact on the resolution of biomarker studies, and provide support for the emerging interest in miRNAs as potential novel and minimally invasive biomarkers for livestock species.

## Introduction

MicroRNAs (miRNAs) are small endogenous RNAs that pair to sites in mRNAs to direct post-transcriptional repression [1]. Recent work indicates that cells release miRNAs in the extra-cellular environment, predominantly in association with either vesicles or protein complexes that protect them from RNases [2]. These miRNAs can be passively released as a result of tissue damage or actively released from healthy cells, from which they may subsequently reach the bloodstream and constitute what it is now referred to as the “blood-circulating extra-cellular miRNome”. Because extra-cellular miRNAs can be easily extracted from any body fluid and profiled through microarrays, real time quantitative PCR or sequencing, blood-circulating miRNAs are currently regarded as being among the most promising clinical biomarkers for the diagnosis, prognosis, and therapeutic treatment of a variety of pathological conditions including cancer, cardiovascular diseases, diabetes, liver pathologies, and sepsis [3]–[5].

Minimally invasive biomarkers which can be profiled by tiny amounts of body fluids are important for animal breeding applications. Livestock species are often subjected to a variety of stress conditions, and extra-cellular miRNAs could be used in tandem with other phenotypic measurements to monitor the responses of individual animals or populations [6]. For example, in human medicine an increase in the abundance of ‘tissue specific’ or ‘organ specific’ miRNAs in blood plasma (or other body fluids) could serve as an indication of toxicity or injury in a particular tissue/organ. Moreover, extra-cellular miRNAs could serve as specific markers for the diagnosis of diseases caused by viruses able to encode miRNAs from their genome (like several herpesviruses), as viral miRNA should be preserved in the extracellular space after the infected host cells die [7].

Intense artificial selection for phenotypic traits of economic importance has produced a large variety of livestock breeds and populations worldwide, and several experimental populations have been raised for research purposes. Among these, two chicken lines have been divergently selected since 1975 for high (R+) or low (R−) residual feed intake (RFI) at constant egg production and body weight, starting from a common unselected population of Rhode Island Red layers produced from six sires and fifty dams [8]. Since then, the two lines have been maintained as closed populations by within-line mating (nine sires and 45 dams per line, with one year generation interval), and the between-line difference in RFI is currently equivalent to five phenotypic standard deviations [9].

The R+ and R− lines differ in only a small proportion of their genome. This has been earlier reported by DNA fingerprinting analyses and interpreted as the combined effect of divergent selection and genetic drift [10]. This appears to be confirmed by preliminary whole-genome sequencing analyses (from pools of seven individuals per line), which identified roughly 850,000 SNPs segregating between the two lines, of which 15,000 are differentially fixed (Lagarrigue et al., unpublished data). Conversely, strong differences between the two lines are found at the phenotypic level. R+ chickens are characterized by higher feed intake and lower adiposity compared to R−, as well as by increased thermogenesis and reduced liver lipogenesis [11]–[13]. An additional striking difference is the excessive appetite in the R+ and the reduced appetite in the R−, which corresponds to an 89% increase in feed intake in the R+ compared to the R−.

Here we evaluated extra-cellular miRNAs circulating in plasma as potential biomarker candidates of a response to a feed deprivation stress in a livestock species. We chose the R+ and R− chicken lines challenged for feed deprivation as an experimental model because of their extreme energy metabolism. Our results indicate that the plasma miRNome of the R+ and R− lines reacts promptly to a feed deprivation stress. This response reflects the phenotypic divergence that these chicken lines acquired through long term artificial selection, implying that even low levels of genetic variation can affect the miRNome considerably. This study is, to our knowledge, the first attempt to characterize the plasma miRNome of a bird species.

## Materials and Methods

### Animals and sampling procedures

All birds were produced and reared under standard conditions at the INRA Experimental Unit PEAT in Nouzilly, France (Pôle d'Expérimentation Avicole de Tours, F-37380 Nouzilly, authorization C37-175-1, 2007) in accordance with European Union Guidelines for animal care, under authorization 37-002 delivered to D. Gourichon (INRA) by the French Ministry of Agriculture. Animal procedures were approved by the Departmental Direction of Veterinary Services of Indre-et-Loire.

Samples obtained for the validation of the sampling procedure: blood samples were obtained from two adult cocks (one R+ and one R−) to extract total RNA from each different blood component (plasma, white cells and red cells) and to check the levels of a subset of miRNAs in each blood component by RT-qPCR (see below).

Samples obtained for deep sequencing and RT-qPCR of miRNAs: nine cocks from the R+ line and nine from the R− line (from five sire families for each line), were blood sampled at 38 weeks of age after 16 hours of feed deprivation (denoted as FD). Five cocks per line were sacrificed before re-feeding to obtain liver samples in the FD condition. The rest of the cocks were blood sampled again four hours after re-feeding (denoted as RF, which represents the control condition) and sacrificed to obtain liver samples in the RF condition. At each blood sampling 2 ml of blood were taken by syringe from the wing vein and gently transferred into EDTA blood tubes. These samples were used for RT-qPCR validations (see below).

In order to obtain sufficient yields of total RNA for the preparation of small RNA libraries, five supplementary cocks per line (belonging to the same sire families as the first sampled group) were blood sampled in the FD and RF conditions. For this group RNAs were extracted from 2 ml of plasma. Six of these samples (three FD and three RF, issued from three cocks per line) were chosen for deep sequencing; the rest of the samples were used for RT-qPCR.

RNA extractions: the blood samples were centrifuged after sampling at 4°C for 10 minutes, at 500 x g; the supernatants were transferred to fresh tubes, centrifuged at 2,000 g x for 30 minutes at 4°C and re-transferred into a new tube with an equal volume of PBS before filtration with a 0.22 µm filter. All RNAs from plasma were extracted using TRIzol LS Reagent according to the manufacturer’s instructions (Life Technologies). Frozen liver samples (about 100 mg each) were homogenized in TRIzol Reagent (Life Technologies) using an Ultra-Turrax (IKA-Werk) and extracted according to the manufacturer’s instructions. RNA yield was assessed using an Agilent 2100 Bioanalyzer and RNA 6000 pico kits. The analyses of small RNA profiles were carried out using the Agilent 2100 expert software (Rev. B.02.08.SI648). RNA samples were stored at −80°C until required for analysis.

### Small RNA library preparation and Sequencing protocol

Small RNA libraries were constructed starting from 25–50 ng of total plasma RNA using the TruSeq SBS Kit v5-GA, while two additional libraries were constructed using the TruSeq SBS v3-HS kit, both from Illumina, according to the manufacturer’s instructions. The quantities of RNA used for small RNA libraries preparation were further checked with a Qubit Fluorometer (Life Technologies). Libraries were sequenced (single read) on a GA-IIx or on a HiSeq1000 Illumina sequencer. The raw reads have been deposited at the European Nucleotide Archive (ENA) with accession number PRJEB6619.

### Post-sequencing analysis

First, a set of unique chicken miRNA precursors was built from the miRBase (version 19) [14] and Ensembl (version 72) databases. While miRBase is composed of experimentally identified miRNAs, Ensembl also includes precursor predictions based on stem-loop structure and sequence homology. The secondary structures of these precursors were then computed using the RNAfold tool from the Vienna RNA package [15]. To assign read count to mature miRNAs, all miRBase mature miRNAs were mapped on the set of precursors without allowing any mismatches. When miRNAs from multiple species, including chicken, mapped at the same position on a precursor, the chicken miRNA annotation was retained. Using this approach we annotated 985 mature miRNAs (including 791 known chicken miRNAs from miRBase and 194 putative orthologs) based upon 1080 non-redundant precursors. For the final set of 41 differentially abundant miRNAs (Table 1), the mature miRNA names were updated using the current miRBase release (version 20).

**Table 1:** Differentially abundant miRNAs in chicken plasma in the six comparisons considered.

miRNAs	Comparisons^a^						
	RF vs FD	R+ vs R−	R+ vs R− (RF)	R+ vs R− (FD)	RF vs FD (R−)	RF vs FD (R+)	Cluster^b^
hsa-let-7a-3p	x						5
gga-let-7a-5p		x		x			3
gga-let-7f-5p	x	x		x	x		3
gga-let-7k-5p		x		x	x		3
gga-miR-19a-3p	x						3
gga-miR-20a-5p		x					3
aca-miR-21-3p		x					3
gga-miR-21-5p	x				x		3
hsa-miR-30c-2-3p	x				x		3
gga-miR-30c-5p	x						5
gga-miR-30d	x						4
gga-miR-31-5p	x				x		3
gga-miR-32-5p	x				x		3
gga-miR-100-5p	x				x		4
gga-miR-101-3p	x				x		3
gga-miR-107-3p		x					3
gga-miR-122-5p		x		x	x		3
gga-miR-126-3p	x						3
ccr-miR-133a-5p		x					3
mmu-miR-144-5p		x					3
gga-miR-184-3p		x		x			3
gga-miR-193b-3p	x				x		1
gga-miR-202-5p		x		x			5
gga-miR-203	x	x		x	x		3
gga-miR-204 (gga-miR-211)	x				x		1
gga-miR-215-5p	x			x	x		3
gga-miR-223	x				x		5
gga-miR-301b-3p	x				x		3
gga-miR-365-3p	x				x		1
gga-miR-499-5p		x		x			3
gga-miR-499-3p		x					3
gga-miR-1736-3p		x	x	x			5
gga-miR-2188-3p		x					1
gga-miR-2188-5p	x				x		3
gga-miR-2954	x				x		1
mmu-miR-143-3p					x		3
mmu-miR-145a-5p	x						1
dre-miR-210-5p	x						5
ENSGALT00000042483-3p		x	x	x			5
ENSGALT00000043002-3p		x					2
ENSGALT00000043002-5p		x					2
**Total**	**23**	**19**	**2**	**11**	**19**	**0**	

a Condition: RF vs. FD; Line: R+ vs. R−; Condition within each chicken line: RF vs. FD (R+) and RF vs. FD (R−); Line within each feeding condition: R+ vs. R− (RF) and R+ vs. R− (FD).

b Cluster number (see Figure 3).

Next, cutadapt v1.2.1 [16] was used to perform 3′ adaptor sequence removal and trim low-quality ends of reads. Reads between 19 nt and 24 nt in length were then collapsed to remove redundancy using an in-house python script. Bowtie v0.12.8 [17] was used to map collapsed reads to the set of chicken precursors, allowing at maximum one mismatch. To discard hairpins with a read distribution inconsistent with Drosha and Dicer processing sites (i.e., reads tilled across the precursor), we expected at least a 3∶1 ratio between reads that matched on any of the stem-loop arms and reads located in the loop. For the remaining hairpins, reads that mapped inside the loop were not considered for quantification. Putative new mature miRNAs were named based upon the name of the hairpin on which they were located when no known mature miRNA matched the same precursor, or from the name of the miRNA mapping on the opposite strand of the precursor. The suffixes “-5p” and “-3p” were added if the new miRNA mapped on the 5′ arm or the 3′ arm, respectively.

Sequences that did not map to any precursor were then successively re-aligned with the same tool and criteria against the *Gallus gallus* genome release 4.0, the RFAM database depleted from the miRNAs, and chicken cDNAs retrieved from the Ensembl database.

### Differential expression (abundance) analyses

The term “differential abundance” was used when referring to miRNAs circulating in plasma. Differential analysis of the small RNA-seq expression data was done using the R/Bioconductor packages edgeR (version 3.2.3) [18] and HTSFilter (version 1.0.0) [19]. Briefly, a negative binomial generalized linear model (GLM) was fit for each miRNA, including a sample-specific normalization factor to adjust for systematic differences among samples due to variable sequencing depth and RNA composition, a fixed line effect (R− and R+), and a fixed feeding effect (FD and RF). As recommended in [19], after estimating per-gene parameters for the full dataset, miRNAs with weak abundance levels across all samples were filtered using HTSFilter, leaving 148 for further analysis. Differential abundance was assessed between lines and feeding groups using a GLM likelihood ratio test, where *P*-values were adjusted for multiple testing using the method by [20] to control the false discovery rate (FDR) at 5%. Log-fold changes of miRNAs identified as differentially abundant in at least one comparison were subsequently visualized using heatmaps. Hierarchical clustering using the Euclidean distance and complete-linkage were used to identify groups of miRNAs based on their log-fold changes from the comparisons previously described in the differential analyses. We note that the interaction contrast between the line and feeding groups was found to be statistically insignificant for all miRNAs.

The analysis of the qPCR data was performed using the R/Bioconductor package limma (version 3.16.5) [21]. For plasma samples, the qPCR abundance data were obtained for each animal from the two feeding conditions (FD, RF), while qPCR data for the liver samples were obtained from different animals in each feeding condition because of the necessity to euthanize the animals for tissue sampling. Using the log_2_-transformed abundance relative to the threshold cycle (CT) value (2^−ΔC^_T_), a linear model was fit for each miRNA. For the plasma data the linear model included fixed effects for line (R− and R+), feeding group (FD and RF), and batch (as plasma samples were collected in two batches), as well as a random effect for each animal to account for the correlation between measurements made on each individual before and after re-feeding. For the liver data the animal random effect and sampling batch effect were omitted because not necessary. Differential abundance was assessed between lines and feeding groups using a moderated t-test, and *P*-values were adjusted to control the false discovery rate at 5% [20]. As before, the interaction contrast between the line and feeding groups was found to be statistically insignificant for all miRNAs in both the plasma and liver.

### cDNA synthesis and qPCR quantification

Reverse transcription was done using miRNA-specific stem loop RT primers [22] and the TaqMan microRNA Reverse Transcription Kit according to the manufacturer’s instructions (Applied Biosystem). In each reaction 10 ng of total RNA from liver or 600 pg of RNA extracted from plasma were mixed with 50 nM specific stem-loop RT primer. RT reactions were carried out at 16°C for 30 min, 42°C for 30 min and 85°C for 5 min. Real-time quantitative PCR was done using standard TaqMan PCR protocols on an Applied Biosystems 7900HT System. PCR primers and probes were optimized to improved stability and mismatch discrimination using locked nucleic acid nucleotides [23] (Table S1).

### Co-abundance analysis of miRNAs

As for the differential analysis (see above), we adopted the term of “co-abundance” when referring to plasma miRNAs. Groups of co-abundant miRNAs were identified from the miRNA-seq data using the clustering approach implemented in the R package HTSCluster (version 1.2) [24]. Briefly, we assumed that the population of miRNAs arises from several distinct subpopulations or clusters, each of which can be modeled separately. The filtered population of miRNAs used for the differential analysis were thus modeled using a Poisson mixture model, where parameter estimation was performed using an Expectation-Maximization algorithm and the appropriate number of clusters present in the data (*K* = 5) was identified using the Integrated Completed Likelihood (ICL) model selection criterion. The cluster-specific parameters may be interpreted as the proportion of total reads attributed to each line (R− and R+) and feeding (FD and RF) combination, which may be useful in identifying global trends in the data.

### Comparison to miRNAs identified in red jungle fowl tissues

We made use of the miRNAs identified by the Chickspress database (available at http://geneatlas.arl.arizona.edu; SRA Bioproject Accession PRJNA204941) to evaluate the relative tissue abundance of differentially expressed miRNAs across a variety of red jungle fowl tissues. The Chickspress miRNA database comprises 659 million single-end, 50 bp reads mapped to the galGal4 version of the chicken genome. miRNA data were collected from two, 20-month-old red jungle fowl (one male, one female) across a variety of tissues: male adrenal gland, male and female adipose tissue, cerebellum, cerebrum, gonad, hypothalamus, heart, kidney, liver, lung, breast-muscle, sciatic nerve, proventriculus and spleen. The database contains mappings to known miRNAs (miRBase v20; [14]) as well as novel miRNAs identified using the miRTrap software [25] for each tissue. A FPKM (fragments per kilobase per million mapped reads) quantitative value is provided for each miRNA feature in each tissue.

### Target gene predictions and functional analysis

An *in silico* search for putative binding sites of differentially abundant miRNAs was performed using TargetScan 6.0 [26]; note that non-conserved miRNAs (ENSGALT00000042483-3p, ENSGALT00000043002-3p, ENSGALT00000043002-5p, gga-miR-1736-3p) and those with borderline differential abundance (adjusted P-values of 0.0498; ccr-miR-133a-5p, mmu-miR-144-5p, gga-miR-20a, aca-miR-499-3p) were not included in the functional analysis. Perl scripts and UTR sequences from 23-species alignments were downloaded from the TargetScan website (http://www.targetscan.org). Predictions were ranked according to the context+ score, which models the contribution of different context parameters on site efficiency including site-type, 3′-pairing, local AU, position, target site abundance, and seed-pairing stability [27]. Only the putative miRNA binding sites belonging to the upper quartile of ranked predictions and present in at least five species including *Gallus gallus* were retained.

The potential target genes for the differentially abundant miRNAs by comparison were then classified according to their biological function determined by the Gene Ontology (GO) System (http://www.geneontology.org). The enrichment analysis to identify over-represented GO categories was performed using the DAVID Functional Annotation Tool (version 6.7; http://david.abcc.ncifcrf.gov). Enrichment was assessed using a modified Fisher’s Exact Test (EASE Score) and P-values were adjusted for multiple testing to control the FDR at 5% [28]. The DAVID clustering feature was used to identify relationships among enriched terms and to cluster them into functional groups.

## Results

### Validation of the sampling procedure

**Figure 1:**
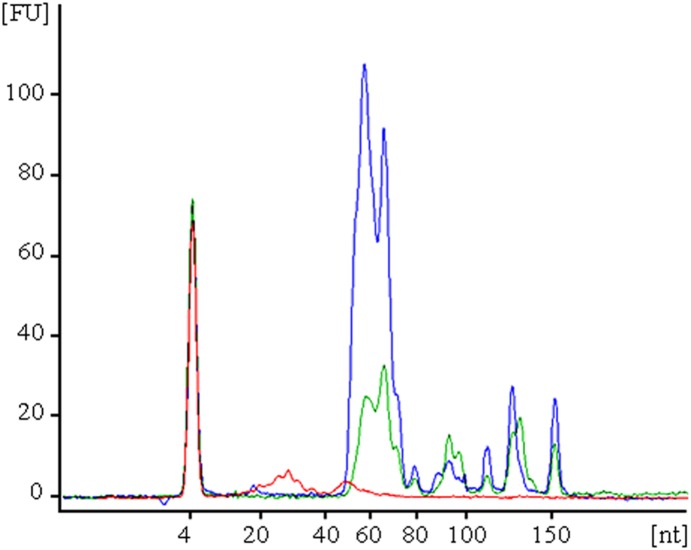
Size and relative abundance of small RNAs isolated from different compartments of chicken blood. Red cells (blue), PBMCs (green) and plasma (red).

In humans, blood cells are a major contributor to circulating miRNA, and factors such as hemolysis can alter plasma miRNA biomarker levels by up to 50-fold [29]. These effects might be exacerbated in birds due to the presence of nucleated erythrocytes. The profiling of small RNAs from chicken blood cell components indicated that small RNAs between 45–55 nt are particularly abundant in red cells followed by a similar peak in PBMCs, and in both these compartments RNAs of larger sizes are detected (Figure 1). In plasma the only RNAs detected are 20–30 nt in length (corresponding to circulating miRNAs), thus suggesting there is no evidence of larger RNA species contaminating our plasma preparations (Figure 1).

### Identification of miRNAs circulating in chicken plasma

**Figure 2:**
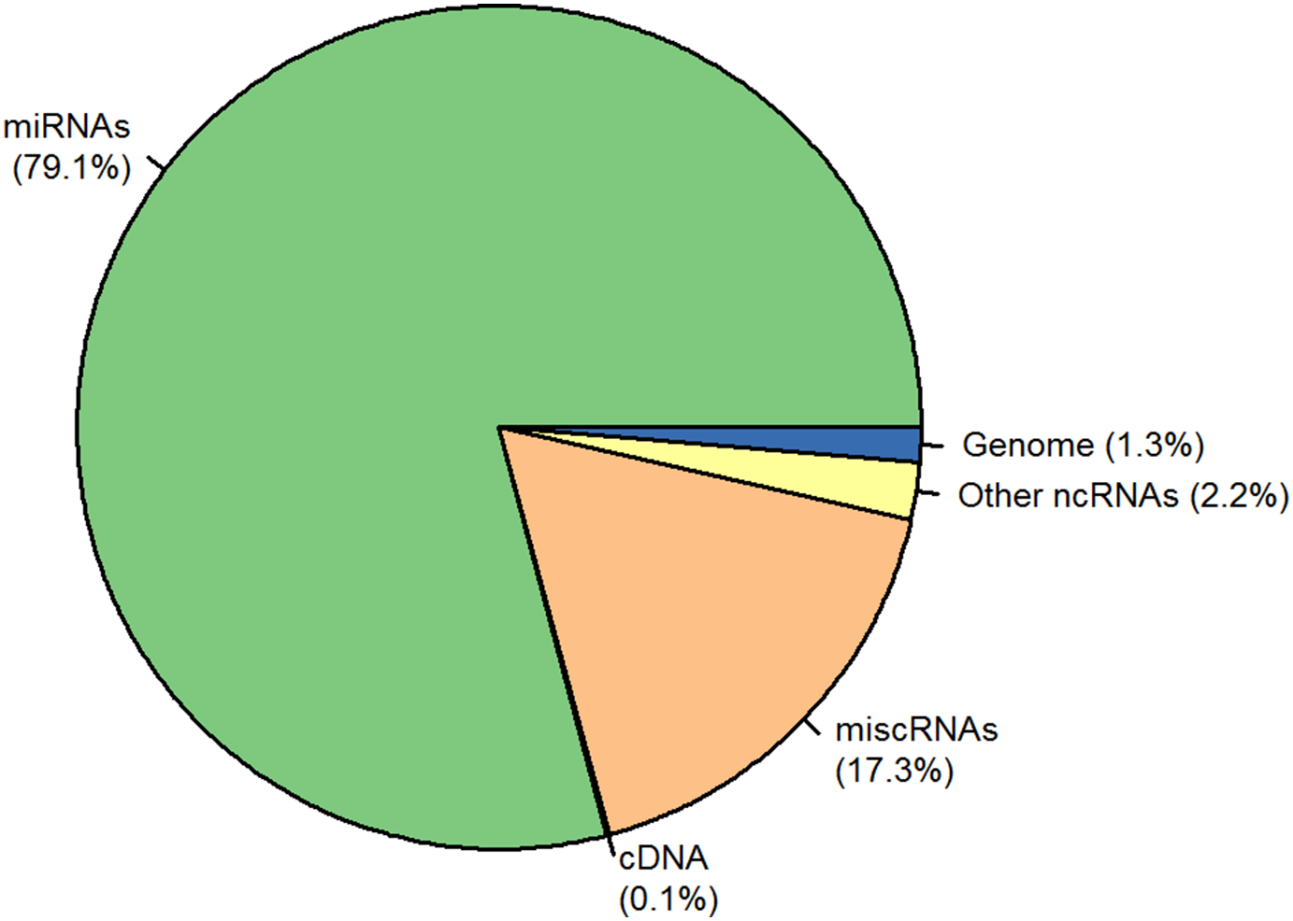
Relative proportions of annotated small RNA-seq reads. Libraries were constructed using total RNA isolated from chicken plasma.

Individual libraries of small RNAs were produced from 3 R+ and 3 R− animals, in both the food-deprivation (FD) and re-fed (RF) conditions (R+ RF; R+ FD; R− RF and R− FD). More than 292 million sequence reads were generated by small RNA-seq. We focused on reads between 19 nt and 24 nt in size which cover the range of sequence lengths for miRNAs. Approximately 45% of the reads were discarded from subsequent analyses, mostly because of their short size (94% of the discarded reads were shorter than 19 nt). The remaining reads were successively aligned to chicken miRNA precursors, the chicken genome, other non-coding RNAs and chicken cDNAs. As expected, most sequences (79%) mapped to miRNA precursors (Figure 2). We did not find any difference in the percentage of small reads corresponding to miRNAs between the different conditions. Finally, 649 mature miRNAs were identified (Table S2). Among these miRNAs, 410 have already been described in chicken, 98 showed a perfect sequence homology with a mature miRNA from another species, 94 were putative new mature miRNAs mapping on the opposite strand of a known chicken miRNA, and 47 miRNAs were Ensembl annotated novel miRNA precursors. The most abundant miRNA was miR-2188-5p, which represented 19% of total reads, followed by gga-miR-10b (gga-miR-10b -5p in miRBase v20), gga-miR-10a-5p, and gga-miR-146c-5p, which together represented about 36% of the total reads (13.3%, 13.9% and 9.2% respectively).

### Effects of genetic divergence and feed deprivation on the plasma miRNAome

#### Differential abundance analysis

The differential abundance analysis of miRNAs circulating in plasma was conducted by comparing the two chicken lines (R+ versus R−) to observe differences between lines (“Line”), and by comparing feed deprived and re-fed birds (FD versus RF) to assess the effect of feed deprivation vs. the re-fed control (“Condition”).

We identified 23 and 19 miRNAs with significant differential abundance in the Condition and Line comparisons, respectively, with only two miRNAs (gga-let-7f and gga-miR-203) found in common between comparisons. The miRNAs representing the Condition contrast were on average more abundantly expressed than those representing the Line contrast (Figure S1).

In addition, we examined the effect of feed deprivation vs. re-fed conditions within each chicken line (“Condition within R+” and “Condition within R−”), which resulted in zero and 19 differentially abundant miRNAs, respectively; the differences between lines within each feeding condition (“R+ versus R− within RF” and “R+ versus R− within FD”) resulted in two and eleven differentially expressed miRNAs, respectively.

The list of differentially abundant miRNAs is reported in Table 1.

#### Clustering analysis of miRNAs

**Figure 3:**
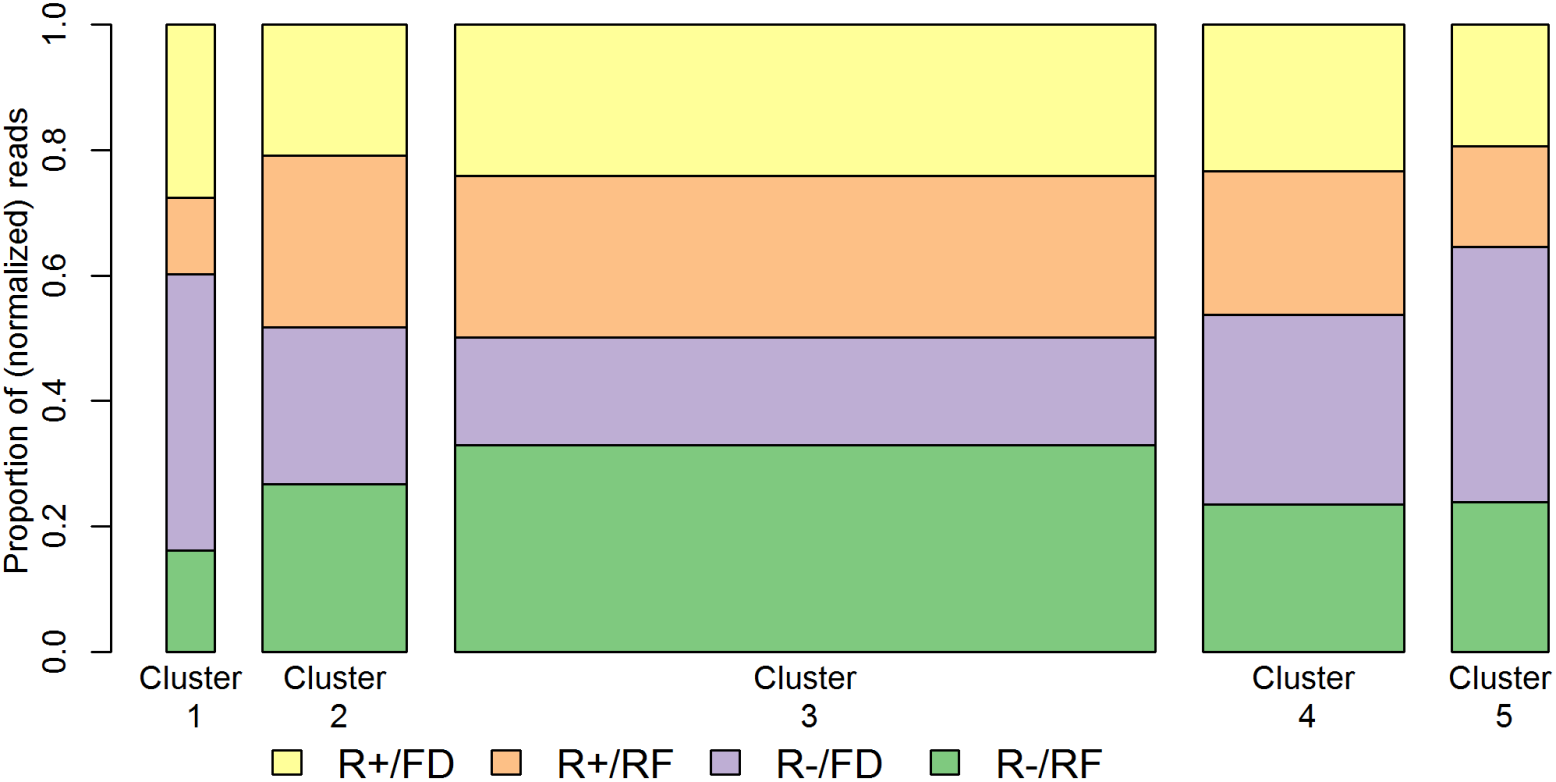
Visualization of five clusters of co-abundant miRNAs in chicken plasma. The analysis was carried out on the 148 miRNAs retained after filtering. FD =  Feed Deprivation; RF =  Re-Feeding.

First, a model-based analysis was carried out on the 148 miRNAs retained after filtering [19] to identify co-abundant groups. Based on the Integrated Completed Likelihood criterion the model with five clusters was retained (Figure 3). The largest cluster (Cluster 3, containing 87 miRNAs) is characterized by under-abundance in R− animals in the feed deprived condition with respect to the other groups. Cluster 1 (containing six miRNAs) and Cluster 5 (containing 12 miRNAs) are largely characterized by over-abundance in the feed deprived condition, particularly for the R− line. Finally, Clusters 2 and 4 (containing 18 and 25 miRNAs, respectively) represent clusters with more balanced abundance among groups. The miRNAs identified as differentially abundant between lines were assigned to Cluster 3 (14 of 19 differentially abundant miRNAs), while those exhibiting differential abundance between feeding conditions were divided among Clusters 1, 3, and 5 (6, 11, and 4 of 23 differentially abundant miRNAs, respectively). Cluster 1 was entirely composed of miRNAs (6 of 6) found to be differentially abundant between feeding conditions (Table 1).

**Figure 4:**
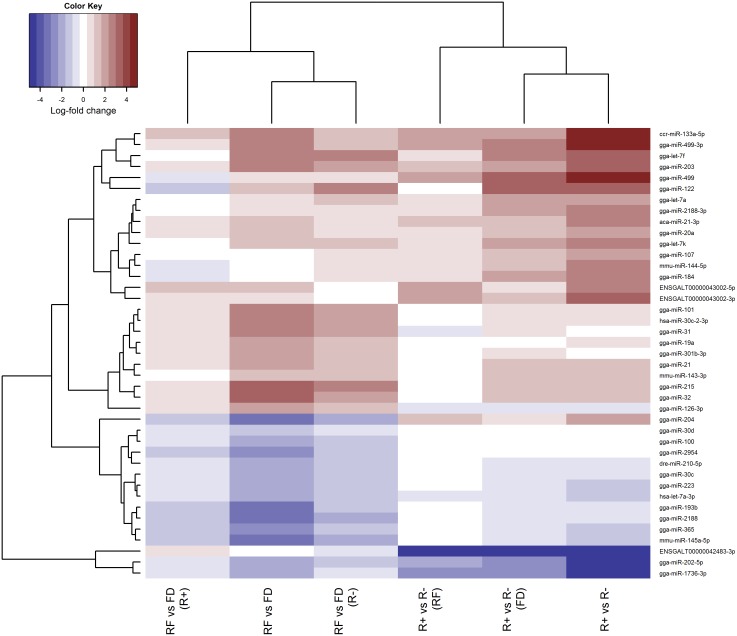
Heat map of log-fold changes of the miRNAs identified as differentially abundant in chicken plasma. Condition: RF vs. FD; Line: R+ vs. R−; Condition within each chicken line: RF vs. FD (R+) and RF vs. FD (R−); Line within each feeding condition: R+ vs. R− (RF) and R+ vs. R− (FD). Red and blue represent over- and under-expression, respectively. Hierarchical clustering has been superimposed on rows and columns.

In addition, we examined a heatmap of the estimated log-fold changes for each contrast for all miRNAs identified as differentially abundant in at least one comparison, where hierarchical clustering was applied to both the miRNAs and the comparisons. This analysis highlighted groups of miRNAs which are either largely over-abundant or under-abundant in the R+ line as compared to the R− line (“R+ vs. R−” column) and groups of miRNAs which are either moderately over-abundant or under-abundant in the RF versus FD groups (“RF vs. FD” column) (Figure 4). The “R+ vs. R− (RF)” and “R+ vs. R− (FD)” comparisons showed that the miRNA plasma content in the re-fed condition is rather similar between the two chicken lines, while larger differences are observed when animals are feed deprived. The comparison “RF vs. FD (R+)” and “RF vs. FD (R−)” indicated that the response to feed deprivation is stronger for the R− line (no miRNAs reached significance in the response to feed deprivation of the R+ line; Table 1).

### qPCR analysis of a panel of differentially expressed miRNAs

#### Plasma

We validated the sequencing results on a larger number of individual birds by RT-qPCR of six miRNAs found to be differentially abundant in plasma. We used plasma samples obtained from eight R+ and seven R− birds, each one sampled in both the FD and RF condition. The miRNAs were selected among those found differentially abundant in the Condition comparison (gga-miR-204, gga-miR-2188-5p and gga-miR-365-3p), in the Line comparison (gga-miR-2188-3p, and gga-miR-122-5p) or in both (gga-let-7f-5p). These miRNAs were found to be present in plasma at very different levels of abundance, from an average of 397 normalized counts (gga-miR-122-5p) to an average of 2.1 million normalized counts (gga-miR-2188-5p).

**Figure 5:**
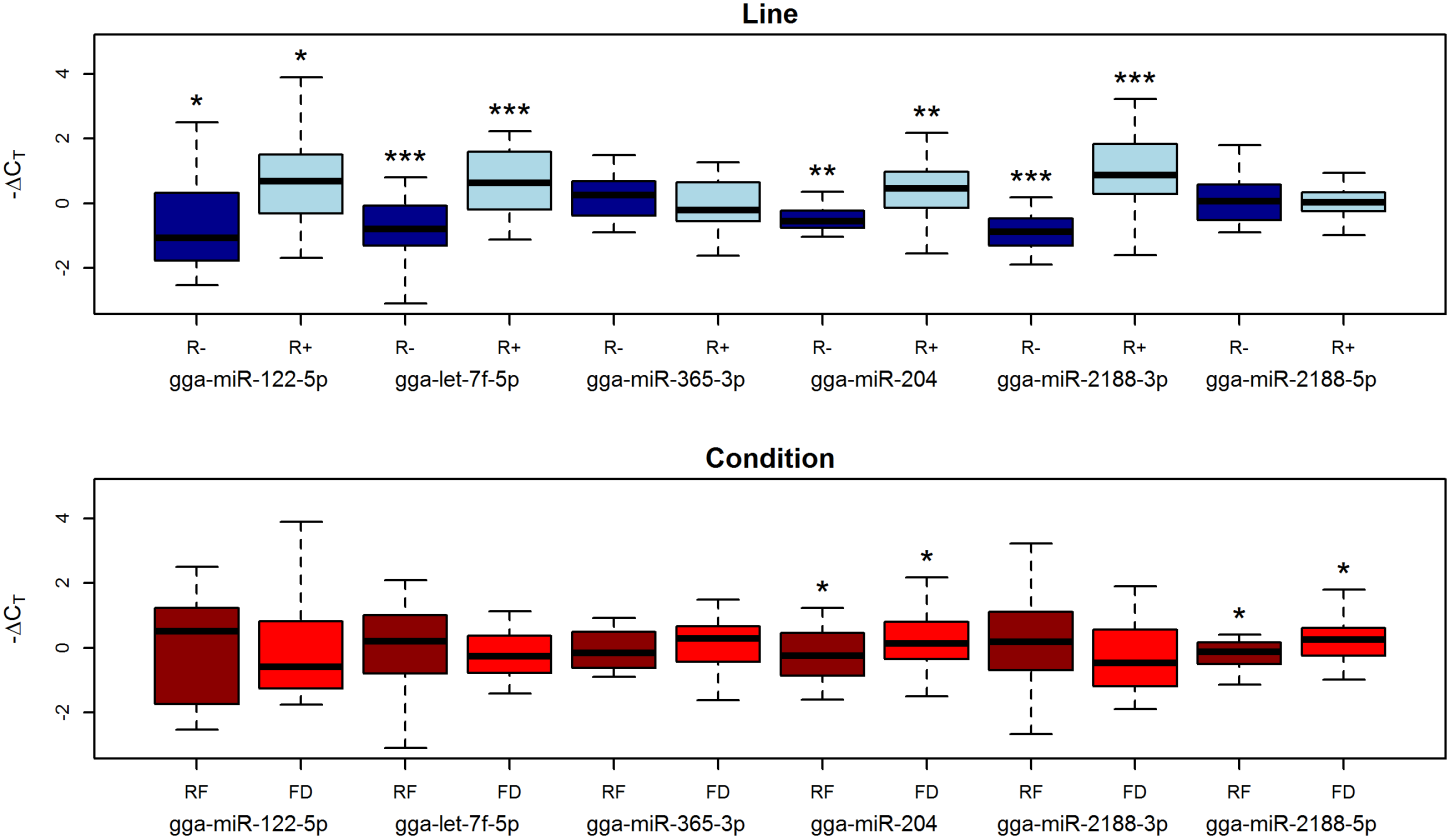
Box-plot (−ΔC_T_ values) of miRNAs profiled by RT-qPCR in chicken plasma samples. Asterisks represent p values: * between 0.1 and 0.05; ** between 0.05 and 0.01; *** <0.01. FD = Feed Deprivation; RF = Re-Feeding.

The RT-qPCR confirmed the relative levels of abundance identified by deep sequencing for all six miRNAs. However, only two (gga-miR-204 and gga-miR-2188-5p) of the four miRNAs that were previously identified as differentially abundant between feeding conditions (Condition) were found to be significant (Figure 5). Conversely, high significance values were found for all miRNAs (gga-miR-122-5p, gga-miR-2188-3p and gga-let-7f-5p) previously identified as differentially abundant between R+ and R- animals (Line). Remarkably, gga-miR-122-5p was confirmed to be significantly more abundant in the R+ animals than in the R− ones despite the high variability observed in the two feeding conditions. Furthermore, gga-miR-204, which was expected to be significant only between feeding conditions, also exhibited significant differential abundance for the Line contrast (Figure 5).

#### Liver

Since the R+ and R− lines differ significantly for metabolic traits such as body fat content and liver lipid metabolism [11], [12], we examined miRNA expression in liver from feed deprived and re-fed animals.

**Figure 6:**
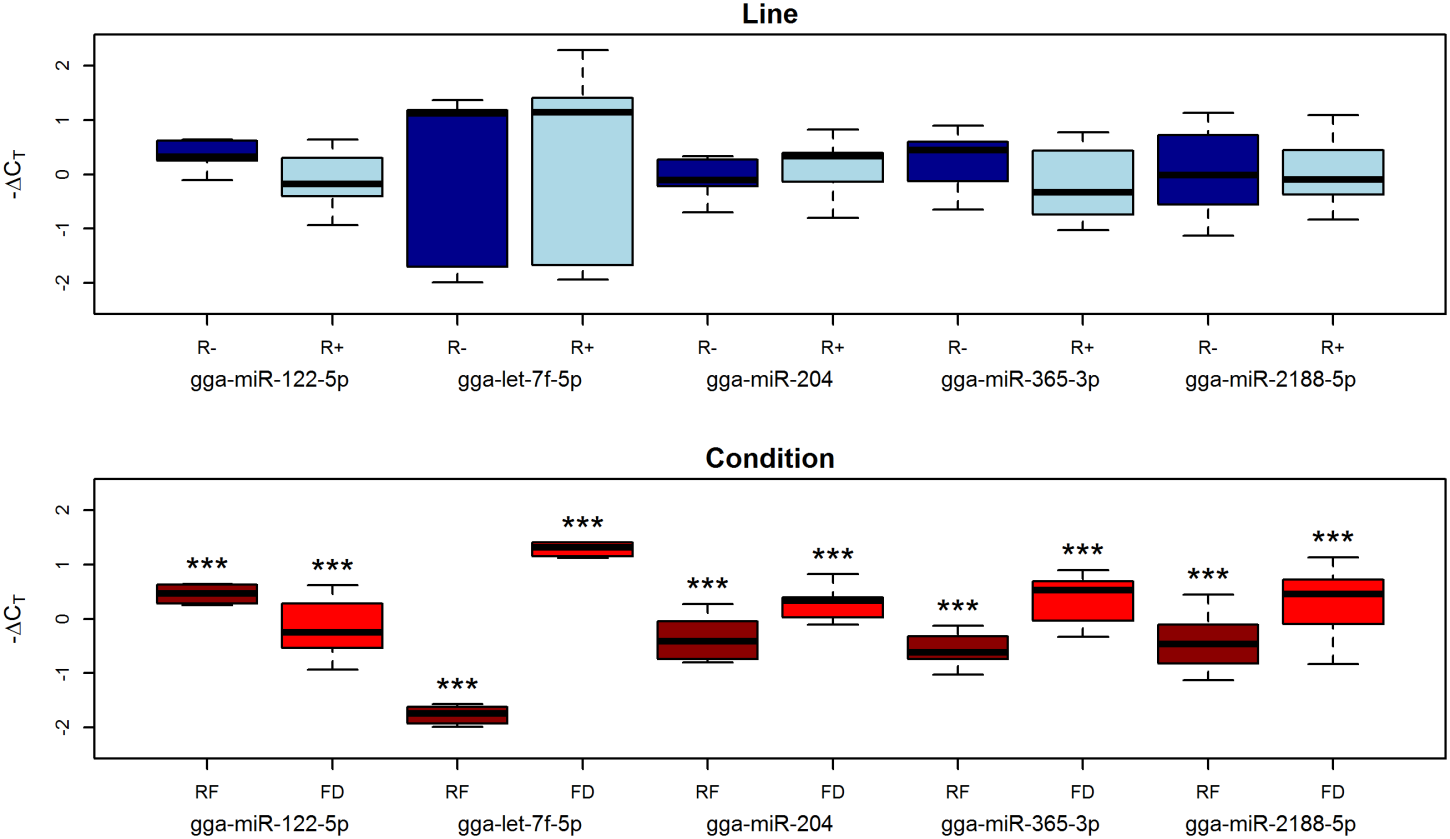
Box-plot of −ΔC_T_ values of miRNAs profiled by RT-qPCR in chicken liver samples. Asterisks represent p values: * between 0.1 and 0.05; ** between 0.05 and 0.01; *** <0.01. FD =  Feed Deprivation; RF =  Re-Feeding.

The analysis showed an opposite pattern compared to that observed in plasma. No significant differences were observed in the Line comparison, while all miRNAs were found to be highly significant in the Condition comparison (Figure 6). With the exception of gga-miR-122-5p, which was significantly downregulated, all miRNAs were strongly up-regulated in response to food deprivation. This pattern suggests that liver miRNAs contribute to the extensive gene downregulation pattern that has previously been observed in the liver of chickens in response to feed deprivation [30].

Interestingly, a significant difference between R+ and R− lines (p = 0.03) was observed for gga-miR-122-5p in the feed deprived condition alone and not in the re-fed one (p = 0.82), with gga-miR-122-5p more expressed in the R− line.

### Conservation and patterns of tissue distribution of differentially abundant miRNAs in plasma

Thirty-five of the 41 mature miRNAs identified as differentially abundant in one or more of the Condition and Line comparisons (Table 1) are encoded by 26 *Gallus gallus* miRNA gene families in miRBase (version 20). This set includes five mature miRNAs (hsa-let-7a-3p, ccr-miR-133a-5p, mmu-miR-144-5p, aca-miR-21-3p and hsa-miR-30c-2-3p), which are present in miRBase as *Gallus gallus* mature miRNAs with a different 3′ editing (Table 2).

**Table 2:** Expression levels (FPKM values) of miRNAs in red jungle fowl tissues.

Comparison	family name	mature miRNA	Adrenal Gland	Adipose	Cerebellum	Cerebrum	Testes	Heart	Hypothalamus	Kidney	Liver	Lung	Breast Muscle	Sciatic Nerve	Proventriculus	Spleen	Blood cells
Line	let-7	gga-let-7a-5p	117335	59675	162187	111094	7131	36021	149480	20644	58028	114420	73053	144277	35454	95840	*
Condition	let-7	gga-let-7a-3p^a^	828	1111	2373	4542	155	694	0	746	847	1250	1185	457	820	1495	*
Common	let-7	gga-let-7f-5p	0	430336	805447	433852	51076	223984	698304	132809	309720	396526	520530	642823	295905	485353	*
Line	let-7	gga-let-7k-5p	54103	86867	177750	411971	0	30906	355820	23045	14685	81346	20127	191737	22353	102206	
Condition	mir-99	gga-miR-100-5p	0	83354	370019	493649	0	21501	424515	63954	147041	113998	12840	180284	49139	39622	*
Condition	mir-101	gga-miR-101-3p	256494	100757	195508	465856	64689	153260	147691	155430	0	208448	258118	88579	182006	461294	*
Line	mir-103	gga-miR-107-3p	17848	11613	109229	150292	2514	7737	91591	5966	21032	9620	2822	9757	12978	7935	*
Line	mir-122	gga-miR-122-5p	247	17627	10	91	49	0	25	4121	870441	441	0	752	332	259	
Condition	mir-126	gga-miR-126-3p	61239	294323	48839	29807	5615	212689	45986	58728	100458	383829	69539	74486	56093	101365	*
Condition	mir-130	gga-miR-301b-3p	17707	13678	56173	140829	9690	19438	58658	19869	13903	29884	8859	6611	23106	27452	*
Line	mir-133	gga-miR-133a-5p^b^	24	16	27	11	0	1964	25	0	0	35	13942	7	22	4	
Line	mir-144	gga-miR-144-5p^c^	0	2305	1059	1476	20	0	1273	1097	3117	11795	0	3297	564	5248	*
Line	mir-17	gga-miR-20a-5p	12836	10555	8846	22925	4153	12154	10698	11096	19706	18149	8458	4792	13034	41208	*
Line	mir-184	gga-miR-184-3p	1110	1651	2523	21225	7077	2457	17319	753	377	7635	756	768	8632	2889	
Condition	mir-19	gga-miR-19a-3p	9333	0	29739	0	12851	12052	0	4626	24231	13171	2616	1237	19222	46565	
Condition	mir-192	gga-miR-215-5p	5432	5289	8038	9551	1460	1763	7909	2668	3207	3079	24083	4030	21442	2444	
Condition	mir-193	gga-miR-193b-3p	14775	18271	4231	3169	118	7489	6807	5826	13944	47733	12367	24001	2563	11878	*
Line	mir-202	gga-miR-202-5p	0	12	60	583	78093	107	46	374	12	0	14	354	30	0	
Common	mir-203	gga-miR-203	203	10	34	324	2671	27	0	167	72	0	0	48	0	14	
Condition	mir-204	gga-miR-204	812	89	3038	855	146	69	3641	82	78	288	67	519	62	522	
Condition	mir-21	gga-miR-21-5p	247016	417933	0	0	0	0	0	0	624785	525619	0	319768	0	1418303	*
Line	mir-21	gga-miR-21-3p^d^	1187	3082	0	0	0	0	0	0	4740	4334	47	1647	0	9366	*
Line	mir-2188	gga-miR-2188-3p	1084	908	716	1593	14	864	744	323	1035	4469	0	1141	150	1377	
Condition	mir-2188	gga-miR-2188-5p	123464	59496	15091	23168	586	45951	22314	22715	68794	404880	0	151890	11073	142447	
Condition	mir-223	gga-miR-223	886	1039	298	501	27	493	477	281	1729	3515	408	840	166	8274	*
Condition	mir-2954	gga-miR-2954	0	0	46445	57839	0	12724	0	0	30671	0	23224	0	0	0	
Condition	mir-30	gga-miR-30c-2-3p^e^	1743	0	1498	3688	276	2248	2145	3061	1053	3476	1788	3473	5772	911	*
Condition	mir-30	gga-miR-30c-5p	55843	85371	108416	131946	7102	57492	106256	70635	81809	84432	98940	42083	163762	60722	*
Condition	mir-30	gga-mir-30d	44213	77276	194808	137192	8062	30389	155052	102122	41951	55163	133273	85033	0	47226	*
Condition	mir-31	gga-miR-31-5p	541	14	2270	1339	5770	0	1154	1119	15	433	0	938	1870	1870	*
Condition	mir-32	gga-miR-32-5p	0	2889	3158	23708	0	2438	4978	3451	8186	4765	2219	1462	5413	13992	
Condition	mir-365	gga-miR-365-3p	1358	9375	573	493	0	1096	416	444	1873	1772	10886	2144	1796	327	*
Line	mir-499	gga-miR-499-5p	11487	440	1873	2682	8160	5640977	417	40	261	557	325	429	66	637	
Line	mir-499	gga-miR-499-3p	261	20	65	27	126	91397	10	1	4	29	18	1	31		
Line	-.	gga-miR-1736-3p	93	93	221	105	11	87	136	59	367	403	174	248	248	237	

*Detected in blood cells by previous studies [45], [46].

a Difference with hsa-let-7a-3p: extra C in 3′.

b Difference with ccr-miR-133a: extra UG in 3′.

c Difference with mmu-miR-144-5p: missing U in 3′.

d Difference with aca-miR-21-3p: extra C in 3′.

e Difference with hsa-miR-30c-2-3p: extra C in 5′.

Three of the six remaining mature miRNAs (mmu-miR-143-3p, mmu-miR-145a-5p and dre-miR-210-5p) have no sequence homology with *Gallus gallus* annotated miRNAs, and three (ENSGALT00000043002-5p and -3p, and ENSGALT00000042483-3p) are encoded by two miRNA genes predicted in the chicken genome (http://www.ensembl.org). ENSGALT00000043002 maps to a non coding region downstream from 5.8 S rRNA, while ENSGALT00000042483 is contained within an intron of a lipoxygenase homology domains 1 gene (*LOXHD1*), and is transcribed in the same orientation. ENSGALT00000042483−3p is the most highly significant miRNA in most differential expression analyses because it was undetected in R+ chickens (Figure 4).

#### Comparison to miRNAs identified in red jungle fowl tissues

The expression of conserved miRNAs across tissues has not been extensively verified in chicken, with only partial data reported so far [31]-[35]. We examined the 35 miRNAs annotated in *Gallus gallus* for their expression levels in 14 tissues of jungle fowl (Table 2). Most of these miRNAs are highly conserved, with conserved patterns of tissue distribution or tissue-specific enrichment. For example, the three members of the let-7 family (let-7a, let-7f, let-7k) are broadly expressed across tissues [36] and tissue enrichment has been found for miR-499-5p and −3p in heart [37], miR-122-5p in liver [38], miR-202-5p in testis [39] and gga-miR-107-3p in brain tissues [40] (Table 2).

The mir-2188 gene is absent in the mammalian lineage. Since its discovery in fish [41] it has been detected in reptiles, amphibians and birds. Both gga-miR-2188-5p and miR-2188-3p are detected in all adult tissues, with the exception of the breast muscle. They are particularly abundant in the lung, and gga-miR-2188-5p is largely predominant across all tissues (Table 2). Gga-mir-2954 and gga-mir-1736 are also absent in the mammalian lineage. The gga-mir-2954 family is avian specific [42]. Gga-miR-2954 has 100% homology with tgu-miR-2954-3p (zebra finch) and is highly expressed in brain (cerebrum, cerebellum), liver, heart and breast muscle, while it is undetected in other tissues.

Gga-mir-1736 has been described only in the chicken [35], [43]. It is ubiquitous in jungle fowl (Table 2), and its low levels of expression across tissues are in agreement with previous findings [35], [43]. This pattern is consistent with the expectation that less evolutionarily conserved miRNA genes are generally expressed at lower levels compared with broadly conserved miRNAs (reviewed by [44]). Interestingly, gga-miR-1736-3p is found in considerable abundance in the plasma of R− animals (approximately 3000 sequence reads), and is six fold less abundant in R+ chickens, independently of the feed deprived or re-fed condition. This miRNA is contained in intron 3 of CARS2 (a mitochondrial aminoacyl-tRNA synthetase encoded by the nuclear genome) and is transcribed in the same orientation.

#### miRNA expression in blood cells

The data of the Atlas support the current view that most organs and tissues release miRNAs in the bloodstream; however, blood cells can be major contributors of the extracellular miRNA content in plasma [2]. Indeed, 19 of the miRNAs found to be differentially abundant in chicken plasma have been reported to be expressed during the different phases of hematopoiesis [45] and T cell development [46] in mammals (Table 2).

As part of our validation of the procedure to obtain chicken plasma miRNAs (Figure 1), we profiled four miRNAs (gga-let-7f-5p, gga-miR-365-3p, gga-miR-2188-5p and gga-miR-2188-3p) by RT-qPCR from whole chicken blood, PBMCs, plasma and red cells. In mouse, the mature miRNAs encoded by mir-let-7f are predominantly expressed in hematopoietic cells, while the homolog of gga-miR-365-3p is ubiquitous and has been detected at low levels in most blood cells [45]. Interestingly, in chicken all four miRNAs are predominantly expressed by red cells and gga-miR-365-3p is undetected in PBMCs (Figure S2). These data are in agreement with the average number of sequence reads found in the plasma of R+ and R− animals for gga-miR-2188-5p (average 2.1 million reads), gga-let-7f -5p (average 143000 reads), gga-miR-365-3p (average 4600 reads) and gga-miR-2188-3p (average 1700 reads). Given that red cells in avian blood are on average 150 times more abundant than PBMCs [47], it can be expected that red cells contribute significantly more than PBMCs to the circulating miRNome of plasma in birds, as our data suggest.

### Target gene predictions and functional analysis

We performed TargetScan predictions to identify the potential gene targets of the conserved differentially abundant miRNAs in the Condition and Line comparisons. As expected, a large number of potential target genes was found (2261 genes, of which 979 were targeted by multiple miRNAs). A great variability in the number of target genes per miRNA was observed, ranging from six for gga-miR-184-3p to 276 for hsa-let-7a-3p.

In the Condition and Line comparisons 145 and 52 significantly overrepresented GO terms were identified, respectively, of which 32 were in common between the two comparisons. Among the most enriched biological functions in both comparisons we found the regulation of DNA and RNA metabolic processes (Table S3, Table S4). To identify functional clusters of overrepresented GO terms, we used the clustering algorithm in DAVID to group similar, redundant and heterogeneous annotation terms. As expected, the top ranked annotation groups for both comparisons included GO terms related to transcriptional regulation and RNA metabolic processes. Moreover, in the Condition comparison, functional clusters with high enrichment scores grouped GO terms related to gene expression and macromolecule biosynthesis and cell morphogenesis (Table S5). In the Line comparison, the top ranked functional clusters included biological processes such as cell motion, vasculature development and epidermis development (Table S6).

## Discussion

The pool of miRNAs circulating in chicken plasma reacts to 16 hours of feed deprivation, with effects that are quickly recovered (4 hours) after re-feeding. Feed deprivation leads to varied changes in abundance of the 148 plasma miRNAs retained after filtering, which grouped into five clusters of co-abundance (Figure 3). However, only the six miRNAs (miR-2954, gga-miR-2188-5p, gga-miR-365-3p, gga-miR-193b-3p, gga-miR-204 and mmu-miR-145a-5p) which compose Cluster 1 are found to be almost three-fold more abundant when concurrently considering R− and R+ animals (Table S2).

Our further results of differential abundance analysis indicate that i) the miRNA response to feed deprivation is indeed strongly influenced by the different genetic backgrounds and that ii) the miRNA divergence between lines is stronger under feed deprived conditions. The net result is that only a limited overlap is found between the sets of differentially abundant miRNAs in the two main comparisons (Table 1). Similarly, a limited overlap was observed in the enrichment analysis of potential target genes, where a broad variety of functional categories was present for both comparisons. This large heterogeneity in the enrichment analyses is expected for miRNAs circulating in plasma. As confirmed by our data (Table 2), plasma miRNAs can originate from several body tissues. This implies that putative targets will necessarily represent several broad GO classes, whose terms cannot be matched *a priori* to the physiology of a specific tissue. An interesting future line of research would be to cross data on the plasma miRNome with a transcriptome analysis from a large variety of tissues from the same animals/conditions to obtain more precise information on the functional impact of circulating miRNAs.

The response to feed deprivation is mostly driven by the response of the R− animals, which appear to be highly reactive to this stress (Table 1, Figure 4). This is reflected by the relatively low significance obtained in RT-qPCR validations in plasma when jointly considering R+ and R− animals (Figure 5). Conversely, the miRNAs that differentiate lines are generally less abundant in the R− animals and this signature is validated even when considering both feeding conditions (Figure 4, Figure 5). However, the difference between the R+ and R− chickens is mostly driven by feed deprivation. Indeed, when animals are fed, only two chicken-specific miRNAs (gga-miR-1736-3p and ENSGALT00000042483-3p) differentiate the two lines, while larger differences are observed when animals are feed deprived, with most miRNAs decreasing in abundance in the R− line (Table 1, Figure 4). From a physiological point of view, similar contrasted responses under fed and feed deprived conditions have been described for these two lines for several plasmatic metabolites [13]. Significant differences were observed only in the feed deprived state for plasmatic concentrations of glucose, non-esterified fatty acids, uric acid, T4 thyroxine hormone and the T3:T4 ratio, implying differences in the control of energy expenditure and the endocrine balance between the two lines, exacerbated in the feed deprived condition [13], [48].

These results can be interpreted in light of the documented physiological divergence that these two lines acquired upon long term divergent selection. The higher feed intake and lower adiposity in the R+ chickens compared to the R− has been justified as a possible alteration of the glucose-insulin axis [13]. This hypothesis is supported by the different plasmatic contents found in the R+ and R− lines of three highly conserved miRNAs with a key regulatory role in energetic metabolism (gga-miR-204, gga-miR-let-7f-5p and gga-miR-122-5p) (Table 2). These three miRNAs are more abundant in the plasma of R+ animals compared to R− (Figure 5) and all respond to feed deprivation in the liver (Figure 6).

Gga-miR-204 has been shown in mammals to be transcribed in the pancreatic beta-cells to block insulin production by down regulating *MAFA,* an insulin transcription factor [49]. In chicken, the plasma levels of gga-miR-204 increase significantly under the feed deprived condition in both chicken lines. This miRNA is detected at low levels in liver (Table 2) where it is strongly upregulated in response to feed deprivation (Figure 6). Together with other members of the let-7 family, miR-let-7f regulates the glucose metabolism in multiple organs [50] and has an important role in the control of fasting glucose concentration [51]. The levels of gga-miR-let-7f-5p in plasma are five-fold decreased in R- animals after feed deprivation, while in the R+ no significant differences in abundance levels were observed (Table S2, Table 1). However, at least in liver, this miRNA is strongly upregulated by feed deprivation (Figure 6). It is remarkable that the three other mature miRNAs encoded by the let-7 family (gga-miR-let-7a-5p and -3p, and gga-miR-let-7k-5p) were found to be more abundant in the R+ than in the R− line (Figure 4, Table S1). Given that in mice the overexpression of the let-7 family leads to decreased fat mass and body weight [50], our data suggest a fundamental role of the let-7 family in response to intense selection for metabolic traits in these lines. Finally, an interesting example is provided by gga-miR-122-5p. This miRNA is known to be implicated in cholesterol biosynthetic pathway and fatty acid metabolism and makes up 70% of all the liver miRNAs [52]. When miR-122-5p is downregulated the hepatic synthesis rate of fatty acids and cholesterol decrease [52], [53], which is in agreement with the finding that this miRNA is downregulated in response to feed deprivation in the liver (Figure 6). However, in plasma, the level of gga-miR-122-5p decreases after feed deprivation only in the R− line. This apparent paradox may be explained by a higher constitutive expression in the R+ line of gga-miR-122-5p by other tissues, with subsequent release in plasma. In particular, in mammals the expression of this miRNA in the adipose tissue has been found to be about 200-fold less than in liver [54] and this is fully confirmed by the jungle fowl Atlas (Table 2). However, this interpretation would contradict the very low amounts of abdominal fat in the R+ animals [11]. Further investigation on other tissues is required to explain these observations, also considering that this miRNA has recently been found to be down-regulated in adipose tissue during diet-induced development of obesity in mice [55].

Our results indicate that even small differences in the genetic background can have a considerable impact on the resolution of biomarker studies. To date most studies of circulating miRNAs have been focused on their clinical relevance as non invasive biomarkers for early diagnosis of disease and monitoring of treatment. To this end, panels of biomarkers need to be ”robust” towards sources of variation due to genetic background, sample processing, age of patients, or other causes [5]. Interestingly, recent studies to define extra-cellular miRNA panels for breast cancer detection revealed differences in miRNA expression between different ethnic groups, with little overlap between Caucasian and African women [56]. Genetic background has been indicated as one of the possible reasons for the ostensible lack of reproducibility in published data regarding circulating miRNAs as markers for breast cancer [57], [58]. Furthermore, it has been reported that a choline- and folate-deficient diet causing nonalcoholic fatty liver disease (NAFLD) determined a different extent of modulation of some miRNAs, including miR-122, in both liver and plasma of divergent strains of mice [59]. These changes in circulating miRNAs based upon genetic variation and diet corroborates the observations in our chicken model.

Overall, this study gives support to the emerging interest on miRNAs as potential novel and non invasive biomarkers for livestock species [60], particularly for the study of phenotypes that reflect the robustness of animals towards environmental challenges in addition to disease. The dynamics of the plasma miRNome upon feed deprivation and recovery reflect the fast kinetics and controlled reversibility of the multi-level transcriptional regulation of stress response, adding evidence to the key role of miRNAs in these processes [61], [62]. It has recently been reported that an Argonaute 2 switch regulates circulating miR-210 to coordinate hypoxic adaptation across anatomically distinct cells [63], adding to the theory that some miRNAs may be specifically secreted in the blood stream and function in intercellular communication between distant tissues [2]. Finally, the observation that the genetic background is an important factor in shaping the extra-cellular miRNome has important consequences for potential field applications. Because the R+/R− lines are an example of extreme phenotypic divergence, it could be expected that the sets of stress-responsive miRNA in plasma will show more overlap between breeds or populations exposed to a stress. However, identifying common panel biomarkers will be challenging in outbred and admixed populations, due to the heterogeneity of genetic backgrounds and to the presence of several sources of environmental variation to which miRNAs are highly responsive concomitantly with other genome regulators [64].

## Supporting Information

Figure S1 **Box-plot of logCPM (counts per million) values of miRNAs found differentially abundant in the Condition and Line comparisons.**
(TIF)

Figure S2 **Expression levels of miRNAs across different blood components.** Data are expressed as mean average Ct values (lower Ct values indicate higher expression) of two biological replicates and three technical replicates. All RT-qPCR were conducted on identical input amounts of total RNA for each blood component.
(TIF)

Table S1 **Sequences of primers and probes used for qPCR validations of six miRNAs.**
(DOCX)

Table S2 **Normalized read counts of 649 miRNAs identified in the plasma of R+ and R**− **samples.** The differentially abundant miRNAs are listed first (in bold), while the rest of the miRNAs are sorted for their average read abundance (last column). Samples from R− line: R06, T05, T09; samples from R+ line: T16, T18, R19. FD =  Feed Deprivation; RF =  Re-Feeding.
(XLSX)

Table S3 **Enrichment analysis of biological processes of the predicted gene targets for the miRNAs differentially abundant in the Condition comparison.**
(XLSX)

Table S4 **Enrichment analysis of biological processes of the predicted gene targets for the miRNAs differentially abundant in the Line comparison.**
(XLSX)

Table S5 **Cluster into functional groups of overrepresented GO terms identified in the Condition comparison.**
(XLSX)

Table S6 **Cluster into functional groups of overrepresented GO terms identified in the Line comparison.**
(XLSX)
