# Supplementary figures and images for: Impact of the Genetic Background on the Composition of the Chicken Plasma MiRNome in Response to a Stress
Source: PLoS One. 2014 Dec 4;9(12):e114598. doi: 10.1371/journal.pone.0114598 (PMC4256448; doi:10.1371/journal.pone.0114598)

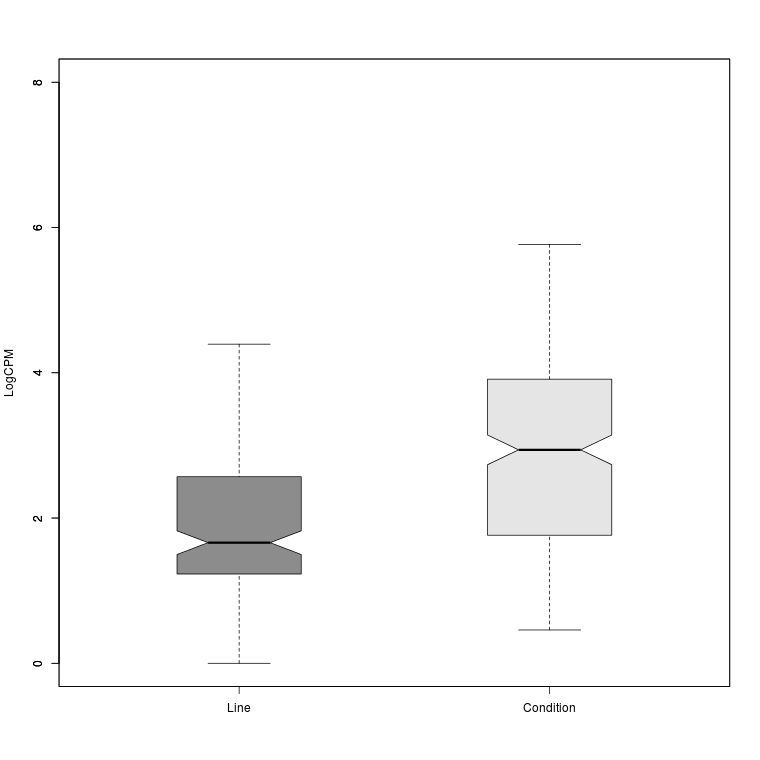

Supplement: Figure S1 — Box-plot of logCPM (counts per million) values of miRNAs found differentially abundant in the Condition and Line comparisons. (TIF) [file pone.0114598.s001.tif]
