# Supplementary material for: Impact of the Genetic Background on the Composition of the Chicken Plasma MiRNome in Response to a Stress
Source: PLoS One. 2014 Dec 4;9(12):e114598. doi: 10.1371/journal.pone.0114598 (PMC4256448; doi:10.1371/journal.pone.0114598)
Supplement: Table S1 — Sequences of primers and probes used for qPCR validations of six miRNAs. (DOCX) [file pone.0114598.s003.docx]

| miRNA Name | Accession | Gene family | Mature sequence | Primer Name | Primer sequence |
| --- | --- | --- | --- | --- | --- |
| gga-miR-204 | MIMAT0003368 | MIPF0000042; mir-204 | UUCCCUUUGUCAUCCUAUGCCU | Stem loop RT primer | GTCGTATCCAGTGCAGGGTCCGAGGTATTCGCACTGGATACGACAGGCAT |
|  |  |  |  | Forwad primer | CGCTTTCCCTTTGTCATCC |
|  |  |  |  | Reverse primer | GTGCAGGGTCCGAGGT |
|  |  |  |  | TaqMan probe | /56-FAM/AT+ACG+AC+AGGC+ATA+GGA/3IABkFQ/ |
| gga-miR-365-3p | MIMAT0003361 | MIPF0000061; mir-365 | UAAUGCCCCUAAAAAUCCUUAU | Stem loop RT primer | GTCGTATCCAGTGCAGGGTCCGAGGTATTCGCACTGGATACGACATAAGG |
|  |  |  |  | Forwad primer | CGCTTAATGCCCCTAAAAAT |
|  |  |  |  | Reverse primer | GTGCAGGGTCCGAGGT |
|  |  |  |  | TaqMan probe | /56-FAM/ATA+CG+A+CA+TAA+GG+ATT/3IABkFQ/ |
| gga_let7f-5p | MIMAT0001162 | MIPF0000002;let-7 | UGAGGUAGUAGAUUGUAUAGUU | Stem loop RT primer | GTCGTATCCAGTGCAGGGTCCGAGGTATTCGCACTGGATACGACAACTAT |
|  |  |  |  | Forwad primer | CGCTTGAGGTAGTAGATTGT |
|  |  |  |  | Reverse primer | GTGCAGGGTCCGAGGT |
|  |  |  |  | TaqMan probe | /56-FAM/AC+GACAA+CTA+T+A+CAA+TC/3IABkFQ/ |
| gga-miR-122-5p | MIMAT0001190 | MIPF0000095;mir-122 | UGGAGUGUGACAAUGGUGUUUGU | Stem loop RT primer | GTCGTATCCAGTGCAGGGTCCGAGGTATTCGCACTGGATACGACACAAAC |
|  |  |  |  | Forwad primer | CGCTTGGAGTGTGACAATG |
|  |  |  |  | Reverse primer | GTGCAGGGTCCGAGGT |
|  |  |  |  | TaqMan probe | /56-FAM/T+GGATA+C+GACA+CA+AA+C/3IABkFQ/ |
| gga-miR-2188-5p | MIMAT0016372 | MIPF0000812; mir-2188 | aagguccaaccucacauguccu | Stem loop RT primer | \|  \| GTCGTATCCAGTGCAGGGTCCGAGGTATTCGCACTGGATACGACAGGACA \| \| --- \| --- \| |
|  |  |  |  | Forwad primer | CGC TAA GGT CCA ACC TCA C |
|  |  |  |  | Reverse primer | GTGCAGGGTCCGAGGT |
|  |  |  |  | TaqMan probe | /56-FAM/ATA+CGAC+AG+GACA+TGTG/3IABkFQ/ |
| gga-miR-2188-3p | MIMAT0027028 | MIPF0000812; mir-2188 | gauauauguggucagaccuauc | Stem loop RT primer | GTCGTATCCAGTGCAGGGTCCGAGGTATTCGCACTGGATACGACGATAGG |
|  |  |  |  | Forwad primer | CGC TGA TAT ATG TGG TCA GA |
|  |  |  |  | Reverse primer | GTGCAGGGTCCGAGGT |
|  |  |  |  | TaqMan probe | /56-FAM/ATA+CGA+CGA+TAGG+TCTG/3IABkFQ/ |
